# Supplementary material for: The ng_ζ1 toxin of the gonococcal epsilon/zeta toxin/antitoxin system drains precursors for cell wall synthesis
Source: Nat Commun. 2018 Apr 27;9:1686. doi: 10.1038/s41467-018-03652-8 (PMC5923241; doi:10.1038/s41467-018-03652-8)
Supplement: Supplementary file 1 — Supplementary Information(PDF 1590 kb) [file 41467_2018_3652_MOESM1_ESM.pdf]

Supplementary Information

The ng_ζ1 toxin of the gonococcal Epsilon/Zeta Toxin/Antitoxin System drains Precursors for Cell Wall Synthesis

Rocker *et al.*

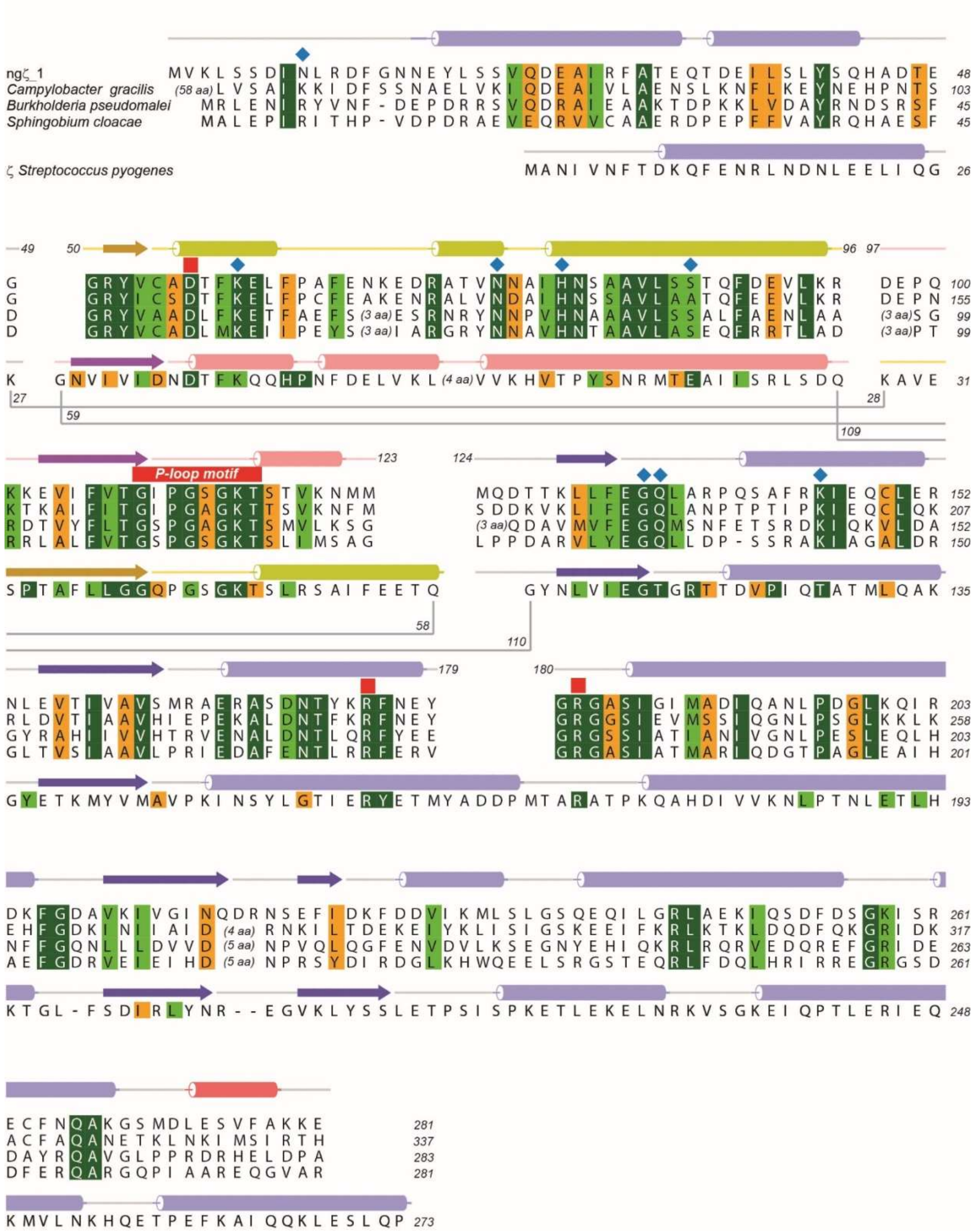

Supplementary Figure 1. **Structure based sequence alignment of ngζ_1 kinase domain with a representative set of homologous proteins and ζ from *Streptococcus pyogenes*.** Sequence conservation of ngζ_1 from *N. gonorhoeae* (NCBI: ADF36622.1), *C. cracilis* (NCBI: WP_005872309.1), *B. pseudomalei* (NCBI: WP_080298172.1), and *S. cloacae* (NCBI: BAV66701.1) was calculated using the AMAS server [1], illustrated using ALSCRIPT[2] and colored from dark green (identical) to orange (homologous). Conserved residues maped on the sequence ζ

from *Streptococcus pyogene*s is adapted from [3]. Secondary structural elements from ngζ_1 as well as from *S. pyogenes* ζ are shown above each sequence block. Helices are illustrated as tons and β-strands as arrows. Gaps that needed to be introduced in the secondary structure are labeled at both sides with the respective residue numbers. Sequence blocks that needed to be swapped in order to match the amino acid sequence of ζ from *S. pyogene*s with that of ngζ_1 are connected by grey lines beneath the sequence and the respective residue numbers how they are connected are given on both sides of the connecting clamp. In addition, the secondary structure elements of the swapped domains are highlighted in green (first domain in amino acid sequence, respectively) and pink (second domain in amino acid sequence). The last helix of ngζ_1 in the sequence alignment belongs to the OB-fold domain and is colored in red. Residues that are important for ATP binding and are conserved in all zeta toxins are highlighted with a red box. Residues that are important for UNAM binding in ngζ_1 are highlighted with a blue diamond.

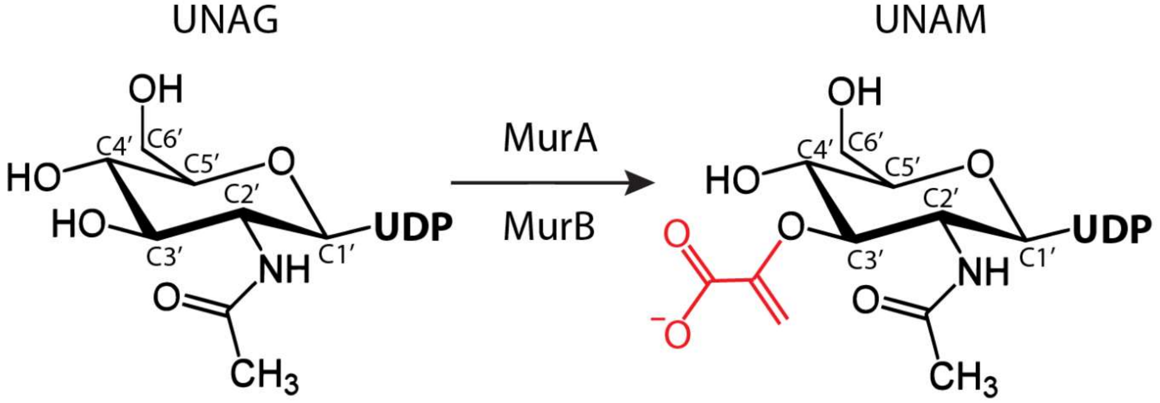

Supplementary Figure 2. **Comparison of UNAG and UNAM.**

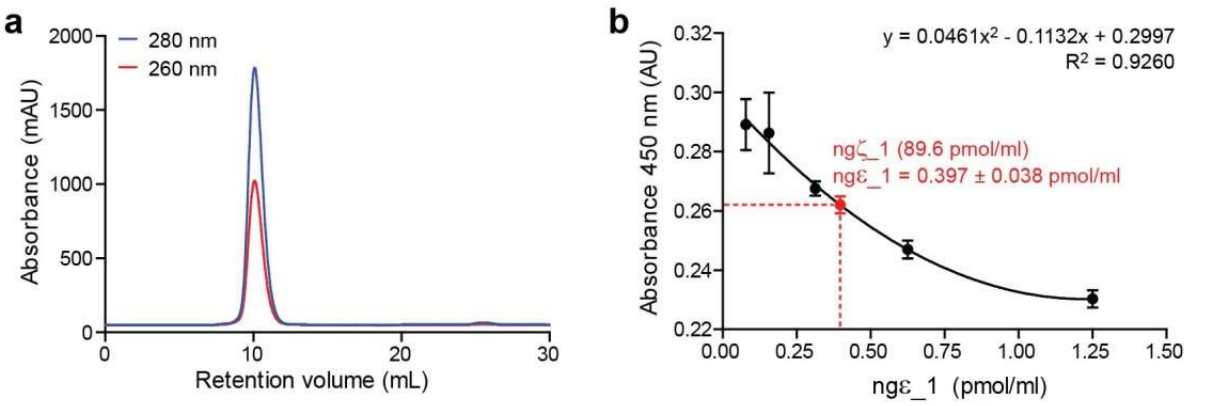

Supplementary Figure 3. Ngζ_1 purification. (**a**) Size exclusion chromatography of ngζ_1 on a Superdex75 10/300 GL column. (**b**) Quantification of residual amounts of ngε_1 in purified ngζ_1 using a His Tag ELISA detection Kit. Standard curve was generated with separately expressed and purified ngε_1 and fitted with a polynomial function of the second degree. Purified ngζ_1 (89.6 pmol/ml) (red) contains approximately 0.44 % of ngε_1 (average of triplicate experiments; error bars indicate standard deviation (s.d.)).

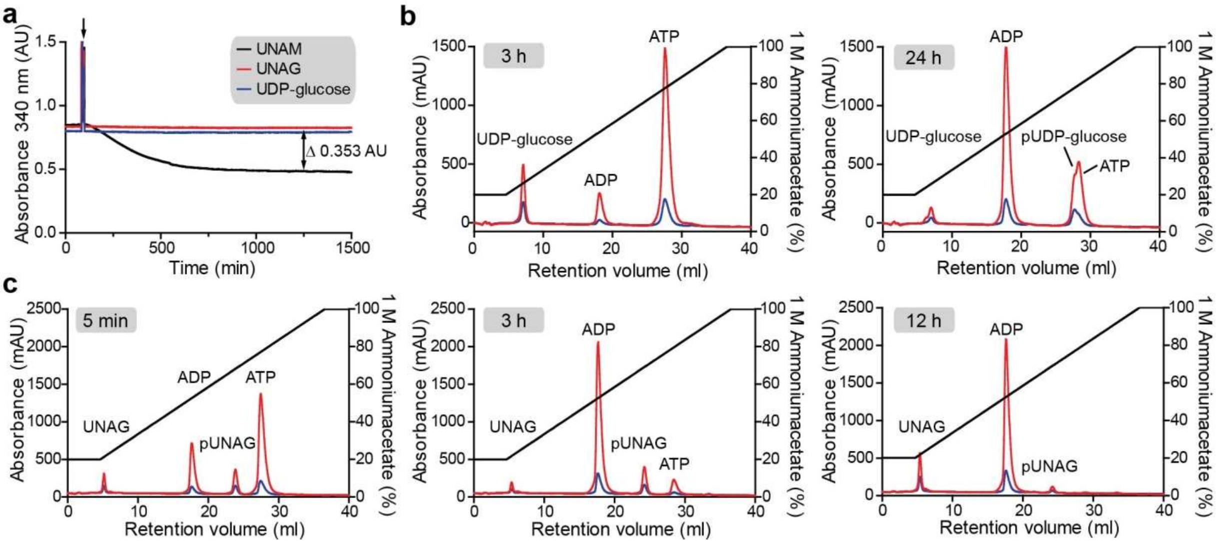

Supplementary Figure 4. Phosphorylation activity of ngζ_1 towards different UDP-sugar species. (**a**) Ngζ_1 activity was determined with a spectrophotometric assay that couples the formation of ADP upon phosphorylation of different UDP-sugar species to the oxidation of NADH. After addition of ATP and ngζ_1 (10 nM) to the reaction a baseline was reached demonstrating the lack of a basal ATPase activity under the used assay conditions. Reactions were started through addition of 50 µM of the respective UDP-sugar substrate (arrow). The decrease in absorbance at 340 nm upon UNAM addition (0.353 AU) corresponds to a reduction in the NADH concentration by 56.8 µM and indicates an equimolar phosphorylation of UNAM by ngζ1. (**b**) Phosphorylation of UDP-glucose (250 µM) by ngζ_1 (1 µM) analyzed via anion exchange chromatography after 3 and 24 h incubation. (**c**) Chromatographic UNAG phosphorylation assay containing 250 µM UNAG and 1 µM ngζ1. (b,c) Absorbance recorded at 260 nm (red) and 280 nm (blue). Retention volumes and the UV 260:280 ratios are indicative for the identity of the respective nucleotide species.

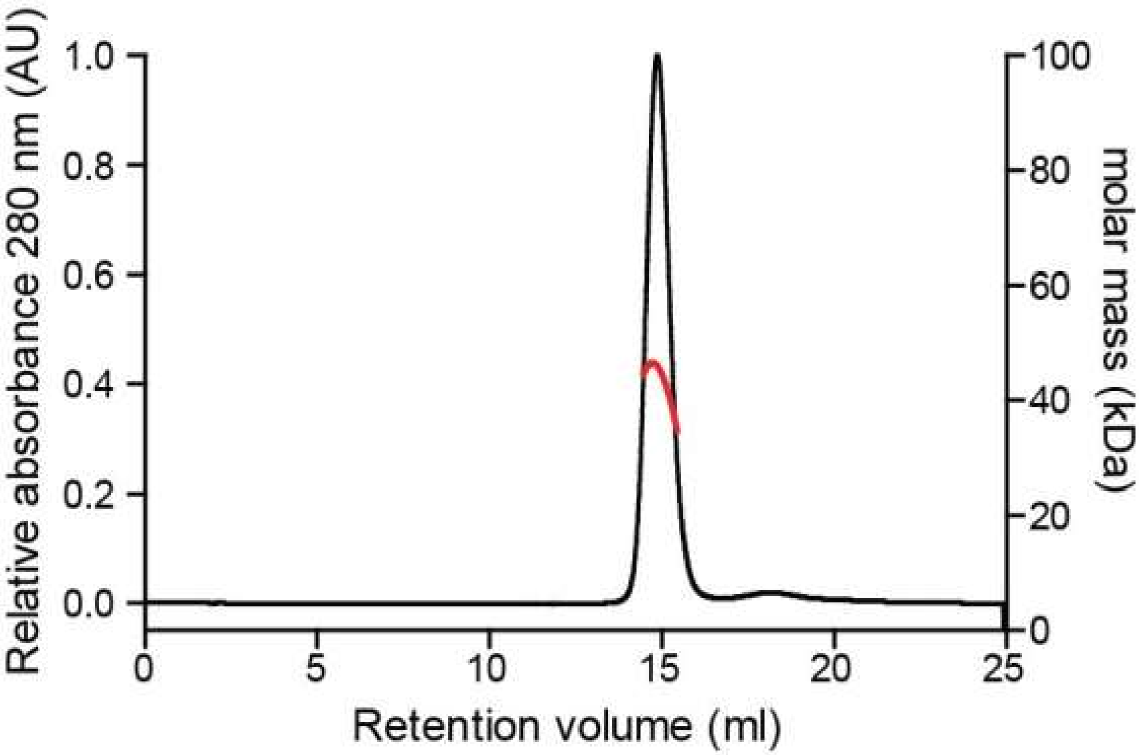

Supplementary Figure 5. Characterization of the ngε_1/ngζ_1 complex. Analytical size exclusion chromatography of the ngε_1/ngζ_1 complex (black) and the molar mass (red) of the eluted species analyzed by multi-angle light scattering. The observed mass corresponds to the calculated molar mass of a heterodimeric complex (ngε_1/ngζ_1: 54.2 kDa, ngε_1: 8.5 kDa, and ngζ_1: 45.7 kDa). (AU, arbitrary units)

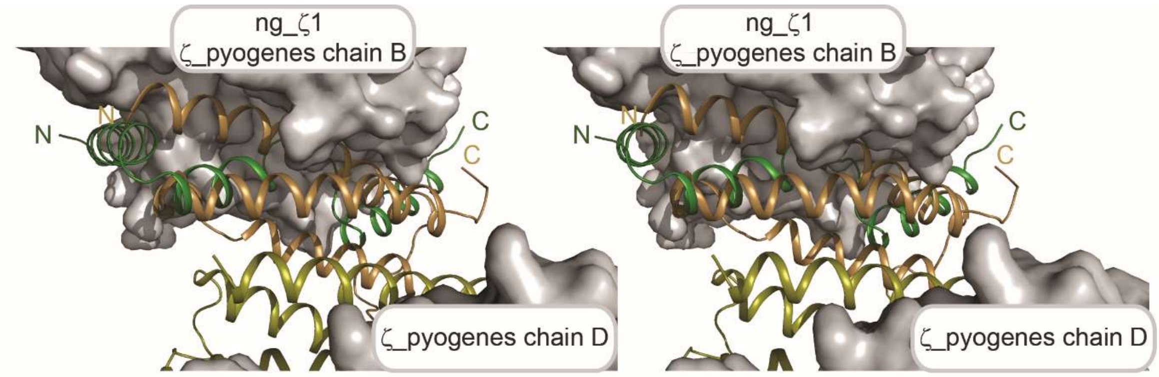

Supplementary Figure 6. Stereoview illustrating the different antitoxin fold of ngε_1 and ε from *S. pyogenes* (1GVN) in complex with the zeta toxin. The ngζ_1 kinase domain of the heterodimeric ngε_1_1/ngζ_1_1 complex is superimposed with one (chain B) kinase protomer of ζ from *S. pyogenes* of the heterotetrameric ε_2/ζ_2 complex. The antitoxins are shown in ribbon representation, where ngε_1 is colored in green, ε from *S. pyogenes* in gold (chain A) and yellow (chain C). For simplicity the toxins are shown as surface representation colored in gray.

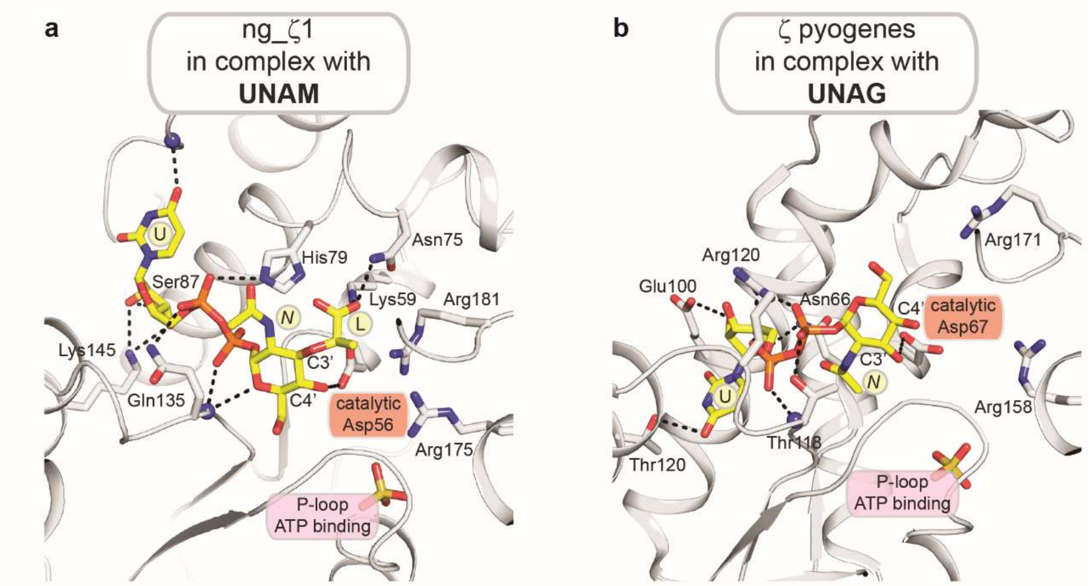

Supplementary Figure 7. Side by side comparison of the active site of ngζ in complex with the substrate UNAM and ζ from *S. pyogenes* (3Q8X) in complex UNAG. The ngζ_1 kinase domain from (**a**) and ζ from *S. pyogenes* (**b**) are illustrated as gray ribbon representation and residues that are important for UNAM or UNAG substrate binding and conserved arginine residues which compensate emerging negative charges during catalysis are shown as stick model. The substrates UNAM and UNAG are shown as stick model with the carbon atoms colored in yellow. Note, that the two substrates are rotated by 180° when compared with each other causing that the C3'-OH and C4'-OH group changed position in the active site. The uracil base moiety (U), the *N*-acetyl (*N*) and the lactoyl-group (L) of UNAM are labelled. Hydrogen bonds are indicated by dashed lines. The P-loop motive important for ATP binding and involved in the phosphoryl-transfer reaction as well as the catalytically important aspartic acid residue that form a hydrogen bond to the hydroxyl group to be phosphorylated are labelled.

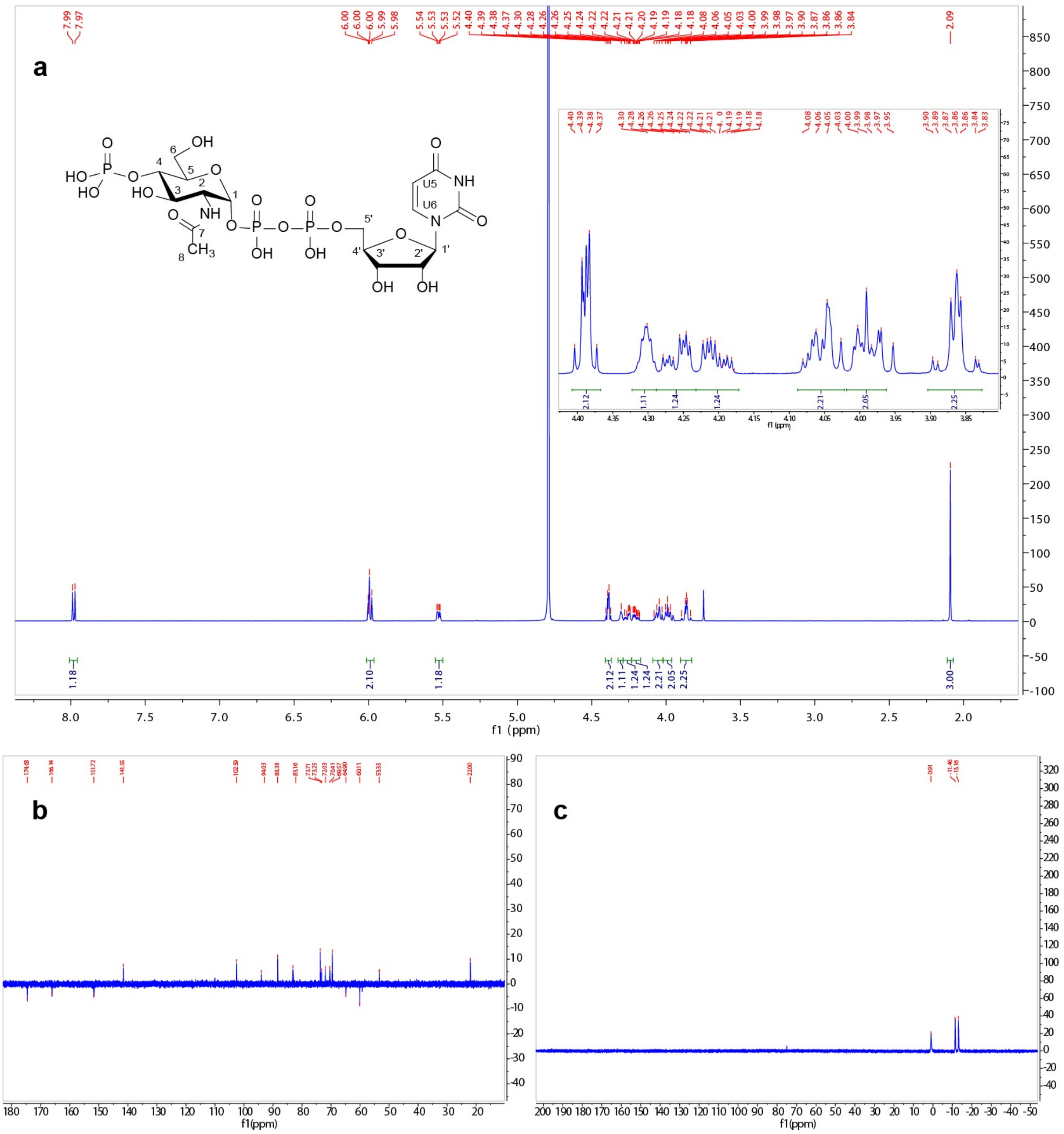

Supplementary Figure 8. NMR spectra of UNAG-4P in D$_2$O; (a) ^{1}H-NMR (500 MHz) with structure of UNAG-4P including atom numbering and zoom-in into the spectra at 4.4-3.8 ppm; **(b)**^{13}C-NMR (152.7 MHz, APT); **(c)** ^{31}P-NMR (202.4 MHz).

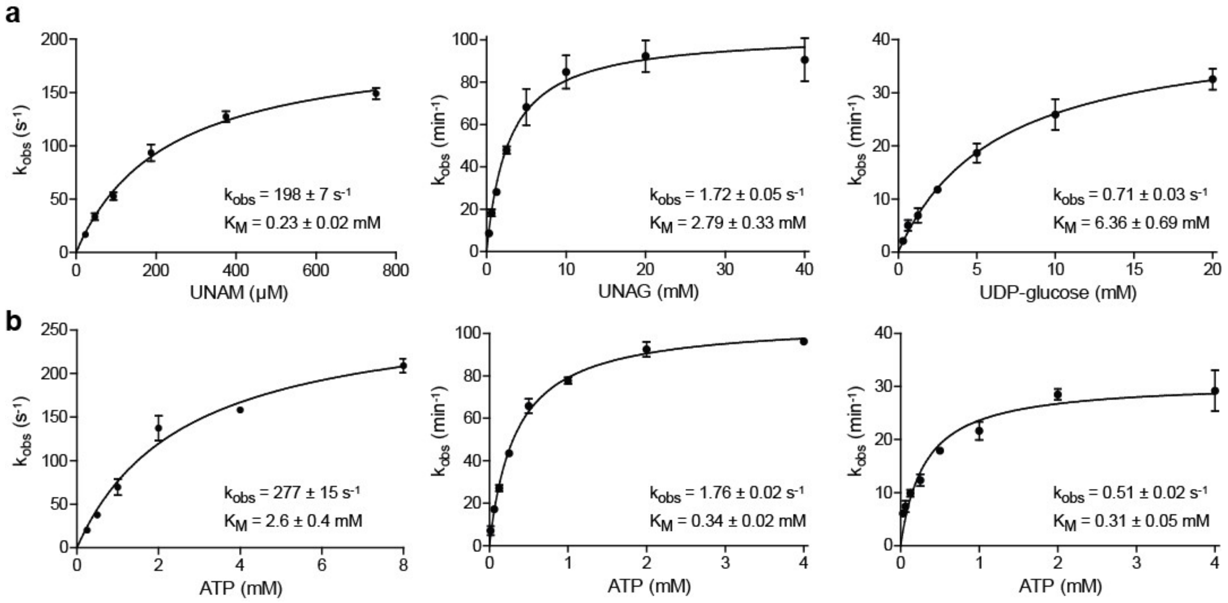

Supplementary Figure 9. Steady state kinetics of ngζ_1 with different UDP-sugars species. (**a**) Ngζ_1 steady state kinetics recorded using 4 mM ATP and varying concentrations of UNAM (left panel), UNAG (middle panel) or UDP-glucose (right panel). (**b**) Steady state kinetics recorded using 1 mM UNAM (left panel), 20 mM UNAG (middle panel) or 20 mM UDP-glucose (right panel) together with varying concentrations of ATP. (Average of triplicate experiments; error bars indicate standard deviation (s.d.))

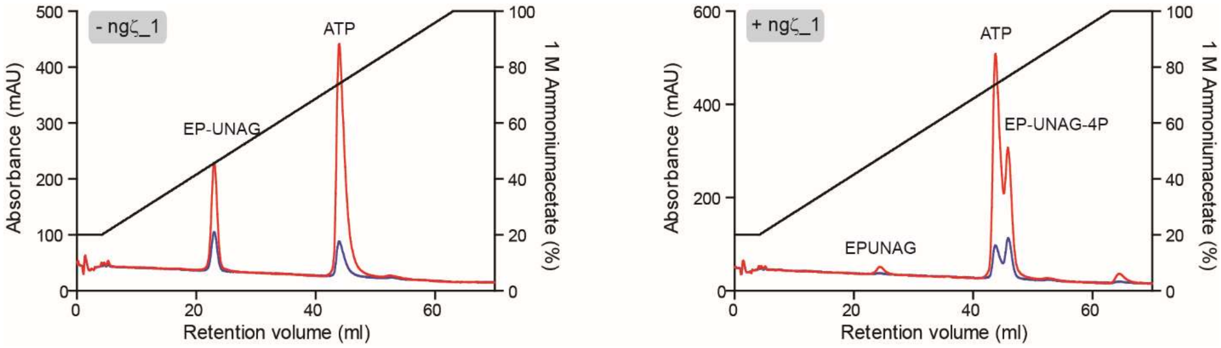

Supplementary Figure 10. EP-UNAG phosphorylation by ngζ1. Chromatographic phosphorylation assay containing 250 µM EP-UNAG and 1 µM ngζ1. Control reaction without ngζ_1 (left panel) and with ngζ_1 after 30 minutes incubation (right panel) analyzed by anion exchange chromatography as described in the Material and Methods section. Absorbance recorded at 260 nm (red) and 280 nm (blue). Retention volume and the UV 260:280 ratio are indicative for the identity of the respective nucleotide species.

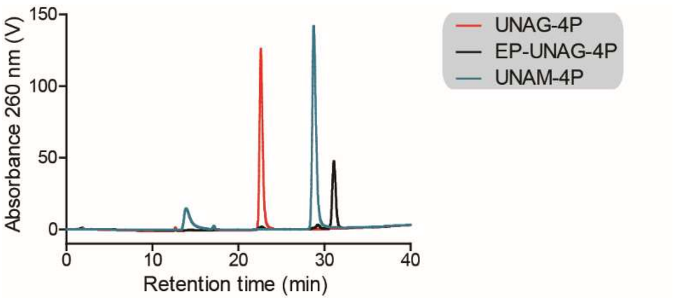

Supplementary Figure 11. Liquid chromatography analysis of ngζ_1 *in vitro* products. Separation of *in vitro* phosphorylated UNAG, EPUNAG and UNAM on a Partisil-5 SAX RACII column as described in the Material and Methods section.

Supplementary Table 1: Proteins with homology to the kinase domain of ng_ζ1 were identified by BLAST and the observed coverage and identity are stated. The sequence homology to the kinase-domain was determined by pairwise sequence alignment with EMBOSS Needle.

species	Accession number	coverage	identity	sequence homology (kinase domain)
Eikenella sp. NML01-A-086	WP_067442134.1	1	0,74	0,852
Campylobacter gracilis	WP_005872309.1	0,98	0,55	0,699
Herbaspirillum sp. GW103	WP_008330209.1	0,9	0,43	0,582
Variovorax sp. KK3	WP_076999118.1	0,92	0,39	0,545
Acinetobacter junii	ENV52027.1	0,92	0,39	0,533
Pseudanabaena sp. 'Roaring Creek'	WP_055074431.1	0,91	0,38	0,54
Alkanindiges sp. H1	WP_076879376.1	0,97	0,37	0,556
Alkanindiges illinoisensis	WP_051526882.1	0,96	0,36	0,552
Dichelobacter nodosus	WP_050707952.1	0,97	0,36	0,563
Stenoxybacter acetivorans	WP_051940839.1	0,92	0,37	0,544
Sphingobium xenophagum	WP_017182896.1	0,92	0,35	0,537
Sphingobium japonicum	WP_037489865.1	0,92	0,34	0,521
Sphingomonas sp. JJ-A5	WP_019053930.1	0,92	0,34	0,521
Sphingobium sp. YBL2	WP_044663217.1	0,92	0,34	0,521
Burkholderia sp. YI23	WP_014194363.1	0,92	0,34	0,521
Novosphingobium sp. KN65.2	WP_054944310.1	0,92	0,34	0,521
Moraxella caprae	WP_029103743.1	0,95	0,38	0,568
Caballeronia udeis	SAL32386.1	0,91	0,36	0,536
Sphingobium cloacae	BAV66701.1	0,92	0,4	0,507
Caballeronia sordidicola	WP_089159908.1	0,91	0,36	0,513
Novosphingobium sp. SCN 66-18	ODU68768.1	0,85	0,39	0,502
Burkholderia pseudomallei	WP_080298172.1	0,92	0,36	0,516
Burkholderia stagnalis	WP_081085665.1	0,92	0,37	0,513
Burkholderia sp. TJI49	EGD05089.1	0,91	0,36	0,502
Caballeronia zhejiangensis	WP_081851911.1	0,91	0,37	0,504
Burkholderia sp. KK1	WP_083747173.1	0,91	0,37	0,504

Supplementary Table 2: Primer. Restriction sites are highlighted in bold and italic font. Mutations are underlined and bold.

Primer name	Sequence 5’ to 3’
ngε_1_f_NdeI	GGC***CATATG***AATAAAGTTGAGCCCCAAGAAAGTAA
ngζ_1_r_HindIII	TTTT***AAGCTT***GATAATCAGCGAAATTTCCGTGATC
ngζ_1_f_K115A	GG***GAATTC***CGGGTTCGGGA**GC**AACTTCGACAG
pET_backbone_f	ATGCGTCCGGCGTAGAGGATC
ngε_1_HindIII	GCC***AAGCTT***ATTGCTCCTTATTTGCCGCCCACAAC
ngζ_1_f_NcoI	CCG***CCATGG***TAAAACTGTCTAGCGATATTAATTTG
ngζ_1_r_SalI	TCCC***GTCGAC***TCTGCTGATGGATTTTTTGGC
ngζ_1_A115K_f	G***GAATTCC***GGGTTCGGGA**AA**AACTTCGACAGTAAAAAACATG
ngζ_1_A115K_r	CATGTTTTTTACTGTCGAAGTT**TT**TCCCGAACCCG***GAATTC***C
MurA_f	ACAAACTAAC***CATATG***GATAAATTTCGTGTTC
MurA_r	C***GAGCTC***TTATTCGCCTTTCACACGCTCAATATTTG
MurB_f	GGTTC***GCTAGC***AACCACTCCTTAAAACCCTGG
MurB_r	CG***GCGGCCGC***ACCGTTCGCTAACAGG
MurC_N2A_f	GCAT***CCATGG***CTACACAACAATTGGCAAAACTGCG
MurC_r	CGAT***GCGGCCGC***GTCATGTTGTTCTTCCTCCGGAG
MurC_A2N_f	ACTTTAAGAAGGAGATATACCATG**AA**TACACAACAATTGGCAAAACTGCG
MurC_A2N_r	CGCAGTTTTGCCAATTGTTGTGTA**TT**CATGGTATATCTCCTTCTTAAAGT

Supplementary Methods

Cloning procedures

E. coli strain DH5α was used for all cloning procedures unless explicitly stated. For primer description see Supplementary Table 2. The initial expression construct pET28b_ngε_1/ngζ_1(K115A) was cloned by making use of the endogenous *Eco*RI site which is located 15 bp upstream of the K115 codon triplet. Two fragments were PCR amplified from pEP5289 (42,004 bp) plasmid DNA (kindly provided by Chris van der Does from the Max Planck Institute for Terrestrial Microbiology, Germany) using the primer pair ngε_1_f_NdeI / ngζ_1_r_HindIII (to obtain the 5' fragment upstream from K115 codon triplet) and ngζ_1_f_K115A / ngζ_1_r_HindIII (to obtain the 3' fragment and introducing an alanine codon triplet by site directed mutagenesis), respectively. After restriction digest, both fragments were consecutively ligated into pET28b plasmid DNA in which the coding region for a thrombin cleavage site was replaced by that for a TEV-cleavage site.

The coding sequence for ngε_1 was amplified from pET28b_ngε_1/ngζ_1(K115A) using the primer pair pET_backbone_f and ngε_1_HindIII and ligated either into pET28b (pET28b_ngε_1) or pBAD/Myc-His A (pBAD_ngε_1) plasmid DNA after *Nco*I and *Hind*III restriction digest. The coding sequence for the mutated ngζ_1(K115A) was amplified from pET28b_ngε_1/ngζ_1(K115A) plasmid DNA using the primer pair ngζ_1_f_NcoI / ngζ_1_r_SalI. After a restriction digest with *Nco*I and *Sal*I the fragment was ligated into linearized pBAD/Myc-His A vector DNA (pBAD_ ngζ_1(K115A)).

For cloning of pET28b_ngε_1/ngζ_1, the mutated K115A codon triplet was back-converted to wild type sequence by QuickChange site directed mutagenesis following the manufacturer's protocol and using the primer pair ngζ_1_A115K_f and ngζ_1_A115K_r. To ensure successful

transformation of the wild-type *ngε_1/ngζ_1* operon, *E. coli* strain C41(DE3) was transformed with pBAD_ngε_1 from which ngε_1 had been expressed prior to preparing chemically competent cells for transformation. Similarly, pBAD_ngζ_1(K115A) was back-converted to wild type sequence (pBAD_ngζ_1) and transformed in the presence of pET28b_ngε_1. After successful cloning, each plasmid mixture was separated by re-transformation into empty DH5α cells and controlled for the loss of the antibiotic resistance conferred by exclusively *ngε_1* gene containing plasmids.

The coding regions for MurA, MurB, and MurC were amplified from chromosomal DNA of *E. coli* strain DH5α using the primer pairs MurA_f/MurA_r, MurB_f/MurB_r, and MurC_(N2A)_f/MurC_r and cloned into pET28b (pET28b_MurA, pET28b_MurB, and pET28b_MurC(N2A)). In case of MurC, the initial clone contained two mutations in the second codon triplet, which was back-converted to the wild type sequence of MurC (pET28b_MurC) by QuickChange site directed mutagenesis following the manufacturer's protocol and using the primer pair MurC_A2N_f / MurC_A2N_r.

Protein purification procedures

Purification of ngε_1/ngζ_1

For ngε_1/ngζ_1 complex purification, the clarified lysate was loaded over Ni-NTA agarose (Qiagen, Venlo, The Netherlands) equilibrated with buffer ngε_1/ngζ_1_Ni (50 mM Tris-HCl pH 8.0, 150 mM NaCl, and 50 mM $(NH_4)_2SO_4$). Bound proteins were eluted with buffer ngε_1/ngζ_1_Ni supplemented with 500 mM imidazole and 2 mM DTE. Protein containing fractions were supplemented with TEV protease for His-tag removal and dialyzed overnight against 50 mM MES-NaOH pH 6.0, 50 mM $(NH_4)_2SO_4$, 0.5 mM EDTA, and 2 mM DTE.

The cleaved His-tag and His-tagged TEV protease were removed by passing the protein solution over Ni-NTA agarose equilibrated with buffer ngε_1/ngζ_1_Ni. The flow-through was diluted with a buffer containing 50 mM MES-NaOH pH 6.0 to a conductivity < 10 millisiemens*cm^{-1} and loaded onto a MonoS 5/50 GL column (GE Healthcare, Little Chalfont, UK) equilibrated with buffer ngε_1/ngζ_1_MS (50 mM MES-NaOH pH 6.0, 50 mM $(NH_4)_2SO_4$, and 2 mM DTE). Bound proteins were eluted in a linear gradient to buffer ngε_1/ngζ_1_MS supplemented with 1 M NaCl (60 CV). Finally, the concentrated protein was polished by size exclusion chromatography using a Superdex75 10/300 GL column (GE Healthcare) equilibrated with buffer ngε_1/ngζ_1_S75 (50 mM MES-NaOH pH 6.0, 200 mM NaCl, and 2 mM DTE). Pure ngε_1/ngζ_1 protein was concentrated and stored at -80 ºC. Selenomethionine-labelled ngε_1/ngζ_1_K115A was purified similar to the native protein, except that the concentration of DTE was increased to 5 mM.

Wild-type ngζ_1 was isolated from the ngε_1/ngζ_1 complex under high salt conditions. Initially, the complex was purified as described before. However, the TEV digest for His-tag removal from ngε_1 during dialysis was omitted. After elution from the MonoS 5/50 GL column, the pooled protein fractions were re-loaded onto Ni-NTA agarose equilibrated with buffer ngε_1/ngζ_1_MS. Ngζ_1 was separated from immobilized ngε_1 by supplementing buffer ngε_1/ngζ_1_MS with increasing concentrations of NaCl (0.15 – 2 M). Subsequently, the ngζ_1 containing flow-through was reapplied four times on a Ni-NTA column to remove any residual His-tagged ngε_1 and the protein was concentrated after the last passage. Finally, the protein solution was applied to a Superdex75 10/300 GL column equilibrated with ngε_1/ngζ_1_S75. Ngζ_1 homogeneity was verified by SDS-PAGE and any residual ngε_1 was quantified to be less than 1 % using the His-Tag ELISA detection Kit (GenScript, Piscataway, USA) following the manufacturer's protocol

(Supplementary Fig.2). Separately expressed and purified ngε_1 was used to generate a standard curve. Purification of His-tagged ngε_1 was performed essentially as described for the ngε_1/ngζ_1 complex. However, in order to avoid ngε_1 protein precipitation additional 150 mM NaCl were included in the dialysis step and the TEV cleavage was omitted.

Purification of MurA, MurB and MurC

The cleared supernatant of a MurA cell lysate was loaded over Ni-NTA agarose equilibrated with buffer MurA_Ni (50 mM Tris-HCl pH 8.0, 200 mM NaCl, and 50 mM $(NH_4)_2SO_4$). The column was washed with high salt buffer (50 mM Tris-HCl pH 8.0, 1 M NaCl), back-equilibrate with buffer MurA_Ni and bound MurA was eluted with buffer MurA_Ni supplemented with 500 mM imidazole. To deplete any potential PEP-MurA adducts, the protein was supplemented with 1 mM UNAG and 1 mM Tris(2-carboxyethyl)phosphine (TCEP) and extensively dialyzed against 50 mM Tris-HCl pH 8.0, 100 mM NaCl, 1 mM EDTA and 0.5 mM TCEP overnight. Subsequently, MurA was bound to a MonoQ 10/100 GL column (GE Healthcare) equilibrated with buffer MurA_MQ (50 mM Tris-HCl pH 8.0, 0.5 mM EDTA, and 2 mM DTE) and eluted in a linear gradient to buffer MurA_MQ supplemented with 1 M NaCl (10 CV). Finally, the pure protein was polished by size exclusion chromatography over a Superdex75 10/300 GL column equilibrated with buffer MurA_S75 (50 mM Hepes-NaOH pH 7.5, 100 mM NaCl, 0.5 mM EDTA, and 2 mM TCEP).

The cleared supernatant of a MurB cell lysate was loaded over Ni-NTA agarose equilibrated with buffer MurB_Ni (50 mM Tris-HCl pH 8.0, 300 mM NaCl, and 5 mM β-mercaptoethanol (β-ME)). The column was washed with MurB_Ni and bound MurB was eluted with buffer MurB_Ni supplemented with 500 mM imidazole. Subsequently, MurB was loaded on a

Superose12 10/300 GL column (GE Healthcare) equilibrated with MurB_S12 buffer (50 mM Tris-HCl pH 8.0 and 100 mM KCl).

The cleared supernatant of a MurC cell lysate was loaded over Ni-NTA agarose equilibrated with buffer MurC_Ni (50 mM Tris-HCl pH 8.0, 500 mM NaCl, and 5 mM β-ME). The column was washed with MurC_Ni and bound MurC was eluted with buffer MurC_Ni supplemented with 500 mM imidazole and 0.5 mM EDTA. Subsequently, MurC was loaded on a Superose12 10/300 GL column equilibrated with MurC_S12 buffer (10 mM Tris-HCl pH 6.7, 500 mM NaCl, and 5 mM TCEP).

Supplementary References

1. Livingstone, C.D. & Barton, G.J. Protein sequence alignments: a strategy for the hierarchical analysis of residue conservation. *Comput Appl Biosci* **9**, 745-56 (1993).
2. Barton, G.J. ALSCRIPT: a tool to format multiple sequence alignments. *Protein Eng.* **6**, 37-40 (1993).
3. Khoo, S.K. et al. Molecular and structural characterization of the PezAT chromosomal toxin-antitoxin system of the human pathogen *Streptococcus pneumoniae*. *J. Biol. Chem.* **282**, 19606-18 (2007).
